# Characterization of NucPNP and NucV involved in the early steps of nucleocidin biosynthesis in *Streptomyces calvus*†

**DOI:** 10.1039/d0ra10878b

**Published:** 2021-01-15

**Authors:** Utumporn Ngivprom, Surayut Kluaiphanngam, Wenjuan Ji, Siriwalee Siriwibool, Anyanee Kamkaew, James R. Ketudat Cairns, Qi Zhang, Rung-Yi Lai

**Affiliations:** School of Chemistry, Institute of Science, Suranaree University of Technology Nakhon Ratchasima 30000 Thailand rylai@sut.ac.th; Center for Biomolecular Structure, Function and Application, Suranaree University of Technology Nakhon Ratchasima 30000 Thailand; Department of Chemistry, Fudan University Shanghai 200433 China qizhang@sioc.ac.cn

## Abstract

Nucleocidin 1 produced by *Streptomyces calvus* is one of five characterized natural products containing fluorine. It was discovered in 1956, but its biosynthesis is not yet completely resolved. Recently, the biosynthetic gene cluster of 1 was identified. The *nucPNP* gene, which was initially annotated as *orf206* and encodes a putative purine nucleoside phosphorylase, is essential for nucleocidin production. In this study, we performed *in vitro* assays and showed NucPNP produced adenine 3 from methylthioadenosine (MTA) 2 and adenosine 4. We also showed the downstream enzyme, NucV annotated as adenine phosphoribosyltransferase (APRT), catalyzes AMP formation from adenine 3 and 5-phospho-α-d-ribose-1-diphosphate (PRPP) 5. However, the catalytic efficiency of NucV was much slower than its homolog *Sc*APRT involved in the biosynthesis of canonical purine nucleoside in the same strain. These results provide new insights in nucleocidin biosynthesis and could guide future research on organofluorine formation.

## Abstract 2

NucPNP and NucV involved in the early steps of nucleocidin biosynthesis were characterized *in vitro*. The results provide insight to study the biosynthesis of nucleocidin in the future.

## Graphical abstract

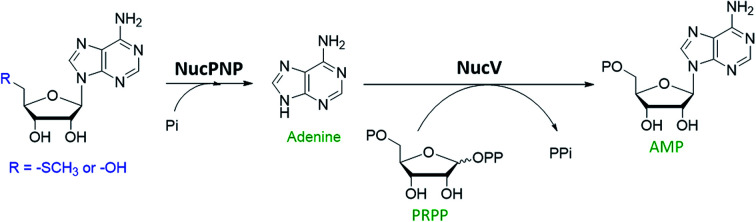

## 1. Introduction

**Fig. 1:**
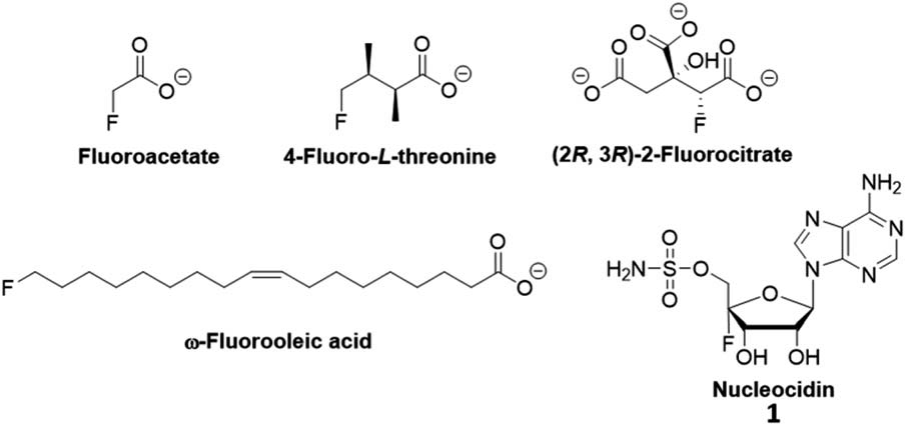
The five characterized natural products containing fluorine.

Organofluorine compounds play important roles in pharmaceuticals,^1^ medical imaging,^2^ materials science,^3^ agrochemicals,^4^ and other disciplines. Due to the interest in fluorination, researchers have searched for natural products containing fluorine. Among an estimated more than 130 000 structurally characterized natural products, there were only five unique fluorine-containing metabolites (Fig. 1) found thus far.^5^ All fluorometabolites, except for nucleocidin, are biosynthesized from 5′-fluorodeoxyadenosine (FDA). The formation of FDA is catalyzed by the only known fluorinase (FldA) in *Streptomyces cattleya*^6,7^ or its homologs in other microorganisms.^8–10^

Nucleocidin was discovered in *S. calvus* ATCC 13382 in 1956.^11^ It is an antimicrobial and antitrypyranosomal analogue of adenosine, which contains a fluorine atom at the C-4′ position of the ribose ring.^12^ Researchers had attempted to investigate its biosynthesis for a few decades without any success. A major reason was that the strain studied could not reproduce nucleocidin. A few years ago, Zechel's group identified a point mutation in the *bldA* gene that is predicted to encode a misfolded Leu-tRNA^UUA^ molecule.^13^ The complementation of *S. calvus* ATCC 13382 with a functional *bldA* gene restored the production of nucleocidin.^14^ At the same time, the genome of *S. calvus* was sequenced and annotated to identify the gene cluster for nucleocidin biosynthesis. Last year, *S. asterosporus* DSM 41452 was identified to be another nucleocidin producing strain after complementation with a functional *adpA* gene.^15^ Its genome sequence revealed a nucleocidin biosynthetic gene cluster nearly identical to that of *S. calvus* ATCC 13382. However, the *fldA* gene or its homolog was not identified in either genome, suggesting nucleocidin biosynthesis involves a different fluorinase from FldA. Feeding experiments with deuterated glycerol suggested that the fluorination step was after ribose biosynthesis.^16,17^ Furthermore, gene knockout experiments for nucleocidin production by O'Hagan's group showed the necessity of *nucGT*, which encodes a glucosyltransferase.^18^ The *in vitro* characterization of NucGT showed that it catalyzed 3′-*O*-β-glucosylation of adenosine and sulfamoyladenosine using UDP-glucose. Furthermore, NucJ, which is annotated as a radical SAM enzyme, was proposed as a fluorinase candidate to catalyze the C–F formation in the biosynthesis of nucleocidin.

Before that, Zechel's group reported the necessity of *nucPNP* (*orf206* in the original paper).^14^ Moreover, the level of mRNA transcript of *nucPNP* increased by 151 times when *bldA* is functional to restore nucleocidin production (Table S1†). In comparison, the mRNA level of *nucGT* only increased by 12 times while that of *nucJ* increased 85 times. Last but not least, *nucV*, which encodes an adenine phosphoribosyltransferase (APRT), showed a similarly increasing level of mRNA transcript (110 times) as *nucPNP*. However, there is a housekeeping APRT in all *Streptomyces* species. According to the *in silico* analysis, NucPNP and NucV are both involved in the biosynthesis of adenosine, which is the core structure of nucleocidin.

**Scheme 1:**
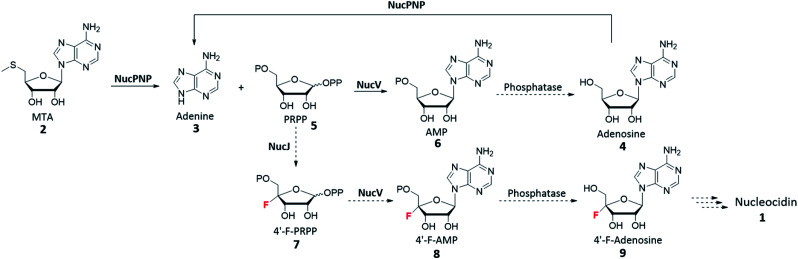
Proposed biosynthetic pathway of nucleocidin 1. The reactions (solid arrow) are studied in this article.

To investigate the functions of *nucPNP* and *nucV*, we heterologously overexpressed these two proteins in *E. coli*. For comparative analysis, *S. calvus* APRT was also expressed. All soluble proteins were purified and characterized by *in vitro* assays. NucPNP converted methylthioadenosine (MTA) 2 or adenosine 4 to adenine 3. NucV catalyzes the synthesis of AMP 6 from adenine 3 and PRPP 5, but its rate was slower than *Sc*APRT. Based on these findings, the biosynthetic pathway is proposed in Scheme 1, which is similar to the biosynthesis of ascamycin and dealanylascamycin.^19^ The substrate of NucV is proposed as 4′-F-PRPP 7 from the NucJ reaction. These results provide clues to solve the mystery of C–F formation in nucleocidin biosynthesis.

## 2. Experimental section

### 2.1 Materials

Chemicals were purchased from either Sigma-Aldrich or TCI Chemicals. Sulfamoyladenosine was synthesized as shown in the ESI.†*Streptomyces calvus* ATCC 13382 was purchased from the American Type Culture Collection. *E. coli* MG1655 was purchased from NBRP-*E. coli* at NIG in Japan. The genomic DNA were purified with a BioFact Genomic DNA Prep Kit. Oligonucleotides were purchased from Integrated DNA Technologies. Enzymes for molecular cloning and the *E. coli* SHuffle T7 Express *lysY* strain were purchased from New England Biolabs. DNA purification kits were purchased from Vivantis. All plasmids made were constructed by Gibson assembly of PCR products.^20^

### 2.2 Overexpression of MBP-NucPNP, NucV, *Sc*APRT, and *Ec*APRT

The gene encoding NucPNP, NucV, and *Sc*APRT were amplified by PCR from the genomic DNA of *S. calvus* ATCC 13382. The gene encoding *Ec*APRT was amplified from the genomic DNA of *E. coli* MG1655. Every PCR product was assembled into pET28 vectors by Gibson assembly.

The NucPNP protein was overexpressed as a fusion with a dual N-terminal 6His/maltose-binding protein (MBP) tag. Soluble MBP-NucPNP was only obtained from expression in *E. coli* SHuffle T7 Express *lysY*. MBP-NucPNP was purified by amylose resin followed by Ni-NTA resin.

The NucV, *Sc*APRT and *Ec*APRT proteins were overexpressed as a fusion with an N-terminal 6-His tag in *E. coli* BL21(DE3). All proteins were purified by Ni-NTA resin.

All information on protein expression and purification is shown in the ESI.†

### 2.3 Enzymatic assays

#### 2.3.1 MBP-NucPNP assays

All assays were performed at room temperature in 50 mM Tris–HCl, 150 mM NaCl, pH 7.5 buffer containing 10 mM potassium phosphate (KPi) and 1 mM substrate, AMP, ADP, ATP, MTA, sulfamoyladenosine, or adenosine. The assays were initiated by the addition of 5 μM MBP-NucPNP. The assays were incubated at room temperature for 2 h and quenched by the addition of trifluoroacetic acid (TFA) to 3% final concentration. The resultant mixtures were analyzed by HPLC (Agilent HPLC 1100) to identify the formation of adenine after phosphorylation. The column and solvent gradient details are shown in the ESI.†

#### 2.3.2 HPLC analysis of NucV and ScAPRT assays

All assays were performed at room temperature in 50 mM Tris–HCl, 150 mM NaCl, pH 7.5 buffer containing 10 mM MgCl_2_, 2 mM adenine, and 500 μM 5-phospho-α-d-ribose-1-diphosphate (PRPP). The assays were initiated by the addition of 5 μM NucV or *Sc*APRT. The assays were quenched by the addition of TFA to 3% final concentration. The mixtures were analyzed by HPLC to monitor the formation of AMP after phosphoribosylation.

#### 2.3.3 NMR analysis of NucV and *Sc*APRT assays

All assays were performed at room temperature in 50 mM Tris–HCl, 150 mM NaCl, pH 7.5 with 20% D_2_O, which contained 10 mM MgCl_2_, 5 mM adenine, and 1 mM PRPP. The assays were initiated by the addition of 10 μM NucV, *Sc*APRT, or *Ec*APRT. The formation of reactions pyrophosphate (PPi) and the consumption of PRPP in the reactions were monitored by ^31^P-NMR (Bruker Avance 500 MHz) at different time points.

### 2.4 Kinetic constant determination

The initial velocity for each substrate concentration was determined by measuring the enzyme activity at different time points in triplicate. The amount of product in the assay was determined by HPLC analysis using the calibration curve of standard, adenine or AMP. The *K*_m_ and *V*_max_ values were calculated by fitting the rate of product formation and substrate concentration in nonlinear regression of the Michaelis–Menten curves with Grafit 5.0 (Erithacus Software, Horley, Survey, UK) shown in Fig. S3–S6.† The apparent *k*_cat_ was calculated by dividing *V*_max_ by the final concentration of enzyme.

#### 2.4.1 The phosphorylation of MTA or adenosine catalyzed MBP-NucPNP

All assays were performed at room temperature in 50 mM Tris–HCl, 150 mM NaCl, pH 7.5 buffer containing 10 mM KPi and varied concentrations of MTA or adenosine. The assays were initiated by the addition of 1 μM MBP-NucPNP. At 15 and 30 min, the assays containing a specific MTA or adenosine concentration were quenched by the addition of TFA to 3% final concentration. The mixtures were analyzed by HPLC to determine the amount of adenine to calculate the initial velocity of reaction.

#### 2.4.2 The apparent constants for PRPP catalyzed by NucV, *Sc*APRT, or *Ec*APRT

All assays were performed at room temperature in 50 mM Tris–HCl, 150 mM NaCl, pH 7.5 buffer containing 10 mM MgCl_2_, 500 μM adenine, and varied concentrations of PRPP. The assays were initiated by the addition of NucV (1 μM), *Sc*APRT (1 μM), or *Ec*APRT (0.1 μM). For NucV, the assays containing a specific PRPP concentration were quenched by the addition of TFA to 3% final concentration at 1 and 2 min. For *Sc*APRT or *Ec*APRT, the assays were quenched at 10 and 25 s. All mixtures were analyzed by HPLC to determine the amount of AMP to calculate the initial velocity of reaction.

#### 2.4.3 The apparent constants for adenine catalyzed by NucV, *Sc*APRT, or *Ec*APRT

All assays were performed at room temperature in 50 mM Tris–HCl, 150 mM NaCl, pH 7.5 buffer containing 10 mM MgCl_2_, 500 μM PRPP, and varied concentrations of adenine. The assays were initiated by the addition of NucV (1 μM), *Sc*APRT (1 μM), or *Ec*APRT (0.1 μM), the assays containing a specific adenine concentration were quenched by the addition of TFA to 3% final concentration at 1 and 2 min. For *Sc*APRT or *Ec*APRT, the assays were quenched at 10 and 25 s. All mixtures were analyzed by HPLC to determine the amount of AMP to calculate the initial velocity of reaction.

## 3. Results and discussion

### 3.1 NucPNP catalysis of phosphorylation of purine nucleoside

**Fig. 2:**
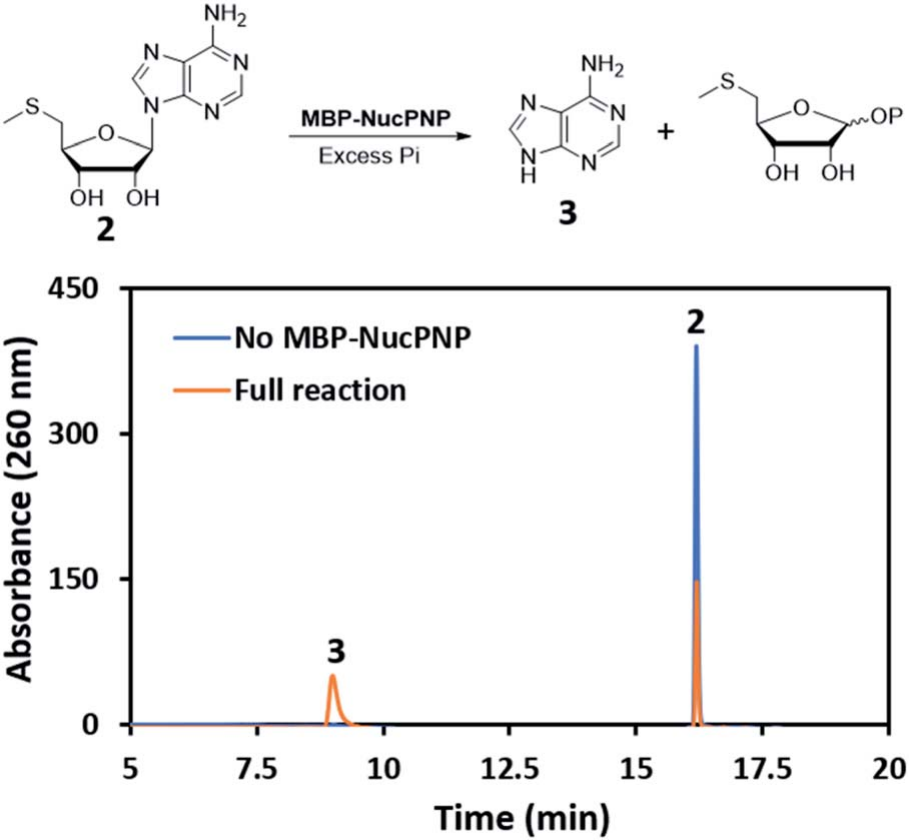
The HPLC analysis of MBP-NucPNP assays to convert MTA 2 to adenine 3.

**Fig. 3:**
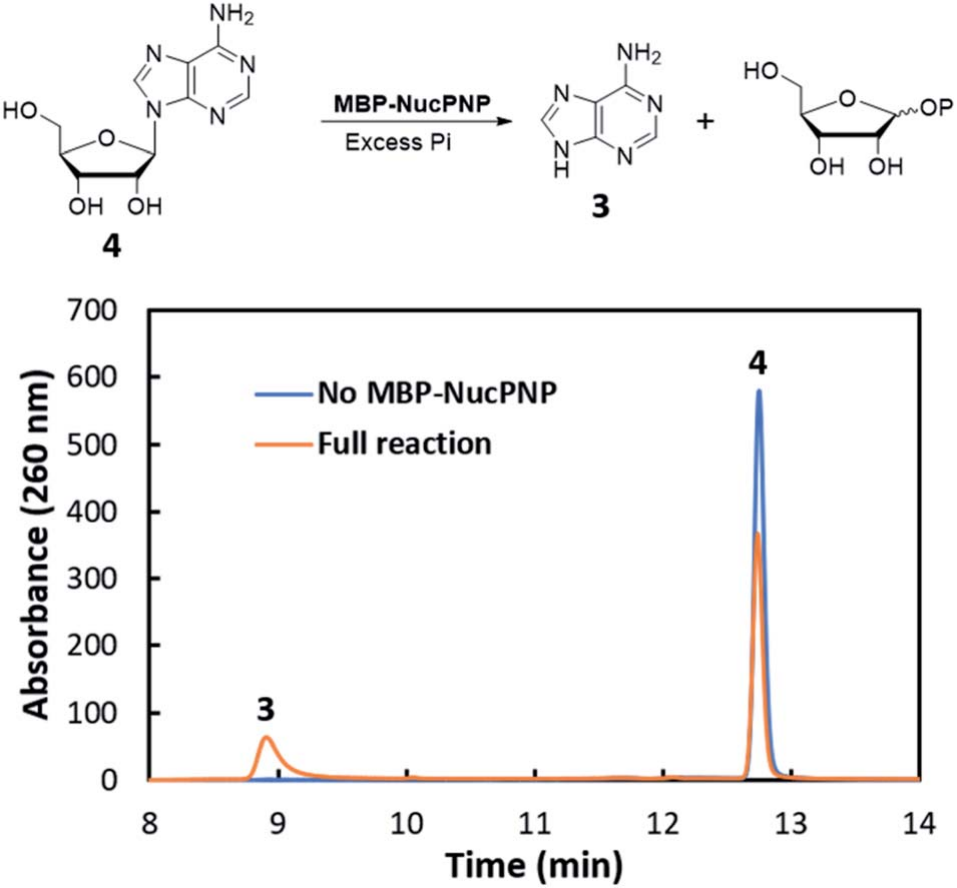
The HPLC analysis of MBP-NucPNP assays to convert adenosine 4 to adenine 3.

NucPNP was annotated as purine nucleoside phosphorylase (PNP).^18^ PNP enzymes catalyze a reversible reaction in purine metabolism,^21^ converting purine nucleoside and phosphate to purine base and α-d-ribose-1-phosphate. In order to identify the substrate of NucPNP, we tried a few different expression systems and soluble tags to obtain soluble protein. Soluble NucPNP only was overexpressed with a fusion with a dual N-terminal His/MBP tag in *E. coli* SHuffle T7 Express *lysY*. It is hereinafter referred to as MBP-NucPNP. Because the *nucPNP* gene is in the nucleocidin biosynthetic gene cluster, a few nucleoside substrates containing adenine, such as MTA 2, adenosine 4, AMP 6, ADP, ATP, and sulfamoyladenosine, were assayed with MBP-NucPNP in presence of excess phosphate. The reaction mixtures were analyzed by HPLC to monitor the formation of adenine 3, which was confirmed by the standard comigration. Among all testing substrates, MTA 2 and adenosine 4 were shown to generate adenine 3*via* phosphorylation compared with the control assays without MBP-NucPNP (Fig. 2 and 3).

**Fig. 4:**
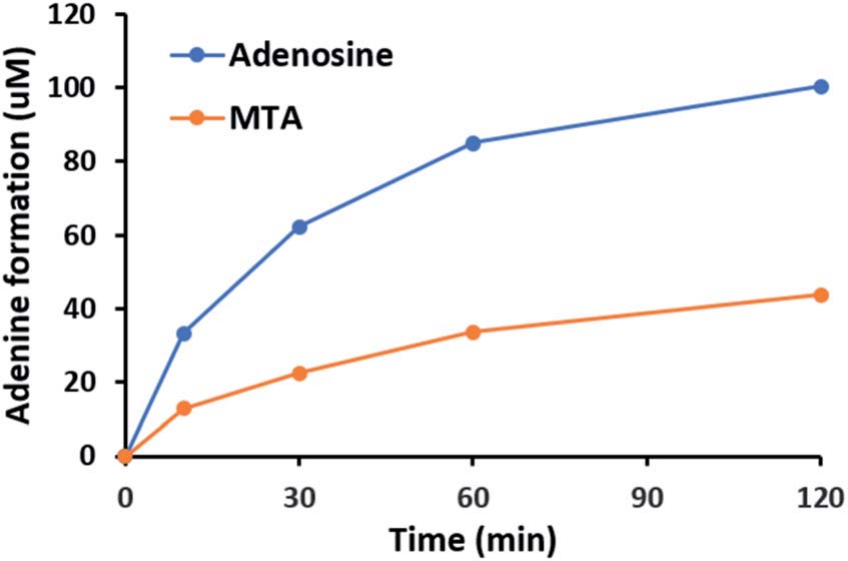
MBP-NucPNP catalyzed the phosphorylation of adenosine 4 to generate adenine 3 faster than MTA 2.

Furthermore, the steady-state saturation kinetic constants of MBP-NucPNP for MTA 2 and adenosine 4 were determined by the formation rates of adenine 3 at different concentrations of MTA 2 or adenosine 4 in the presence of 10 mM of phosphate (Table 1). The apparent *k*_cat_ of MTA 2 is about three-fold lower than adenosine 4 and the apparent *K*_m_ of MTA 2 is half of adenosine 4. Moreover, the time course experiments were performed at 200 μM adenosine 4 or MTA 2 in the presence of excess phosphate to undergo the phosphorylation at the rate of *V*_max_. The results showed that the formation of adenine 3 from adenosine 4 was more than MTA 2 in the period of reaction (Fig. 4). Moreover, the amounts of adenine 3 formation from adenosine 4 were about 2.5 folds higher than MTA 2, which was similar to the ratio of their apparent *k*_cat_ values.

**Table 1:** The kinetic constants of MBP-NucPNP for MTA and adenosine^a^

Substrate	*k*_cat_ (s^−1^)	*K*_m_ (μM)	*k*_cat_/*K*_m_ (μM^−1^ s^−1^)
MTA	0.15 ± 0.01	43.5 ± 4.1	3.4 × 10^−3^
Adenosine	0.49 ± 0.02	90.3 ± 8.2	5.5 × 10^−3^

a The *k*_cat_, *K*_m_, and *k*_cat_/*K*_m_ were determined at a fixed concentration of 10 mM phosphate with varied MTA or adenosine catalyzed by 1 μM of MBP-NucPNP.

In adenosine metabolism, MTA 2 or adenosine 4 could be metabolized to form adenine 3 by 5′-methylthioadenosine phosphorylase or adenosine phosphorylase, respectively.^21^ Similarly, adenine 3 could be utilized to biosynthesize nucleocidin 1 (Scheme 1). In natural product biosynthesis, a similar reaction was reported in aristeromycin biosynthesis.^22^ Its MacI and MacT enzymes were characterized as adenosine phosphorylases. Their functions were proposed to generate adenine and catalyze an irreversible reaction for the biosynthesis of neplanocin A and aristeromycin.

Moreover, to investigate the possible self-resistant function of NucPNP, sulfamoyladenosine, a nucleocidin analogue without fluorine at the C-4′ of ribose, was synthesized and assayed in the phosphorylation condition (Fig. S2†). We did not observe any conversion by HPLC, which suggested that NucPNP does not act on sulfamoyladenosine (and likely nucleocidin).

According to the *in vitro* characterization, the reported increase in the mRNA level upon complementation to allow nucleocidin synthesis, and the gene knockout experiments,^14^ NucPNP is proposed to be an essential enzyme to generate adenine for nucleocidin biosynthesis.

### 3.2 The NucV-catalyzed AMP formation is slower than those by *Sc*APRT and *Ec*APRT

Although NucPNP is proposed to have a function to generate adenine 3 from MTA 2 or adenosine 4, adenosine 4 is a precursor to biosynthesize sulfamoyladenosine. It was proposed as an intermediate in the biosynthesis of dealanylascamycin,^19^ a nucleocidin analogous. In addition, in the purine nucleoside metabolism, adenosine 4 could be synthesized from adenine 3 and PRPP 5 to form AMP 6 in an APRT-catalyzed reaction,^23^ followed by dephosphorylation. In the gene cluster of nucelocidin, *nucV* was annotated as an APRT. Therefore, we raised a question why NucPNP could phosphorylase adenosine 4 back to adenine 3. Moreover, the kinetic experiments of MBP-NucPNP also showed that MBP-NucPNP had 1.6-fold higher *k*_cat_/*K*_m_ for adenosine 4 than MTA 2 (Table 1). It suggested that MBP-NucPNP slightly prefers adenosine 4 as the substrate. Therefore, we hypothesized that the possibility of fluorination occurs between the NucPNP and NucV reactions. In the fluorometabolite biosynthesis in *S. cattleya*, FlB was shown to selectively phosphorylase 5′-fluoro-5′-deoxyadenosine faster than adenosine.^24,25^ It made us hypothesize that 4′-F-PRPP 7 might be the substrate of NucV. In our scenario, NucV could convert 4′-F-PRPP 7 and adenine 3 to form 4′-F-AMP 8 faster than PRPP 5. Unfortunately, 4′-F-PRPP 7 is not available from any source.

Therefore, we used a bioinformatic approach to evaluate our hypothesis. We searched the amino acid sequence of NucV against the genome of *S. calvus* by BLAST. The results showed that *S. calvus* has one NucV homolog (hereafter named as *Sc*APRT) with 79% similarity and 62% identity. Moreover, both amino acid sequences have the PRPP binding site motif.^26^ We further blasted the *Sc*APRT amino acid sequence against all *Streptomyces* species. *Sc*APRT is a housekeeping APRT for nucleoside biosynthesis. Hence, we compared the reaction rate of *Sc*APRT and NucV to gain more information. Soluble *Sc*APRT or NucV with a N-terminal His tag were expressed in *E. coli* BL21(DE3) and purified by affinity chromatography. Both enzymes were tested to catalyze the formation of AMP 6 from PRPP 5 and adenine 3 in the presence of MgCl_2_ by HPLC analysis.^26,27^ Since APRT enzymes were reported to catalyze reversible reactions,^27^ the backward reactions were tested by incubating AMP 6 and excess pyrophosphate (PPi) with each enzyme. No adenine 3 formation was observed in HPLC and ^31^P-NMR analysis (data not shown). It suggested that both enzymes favour the forward reaction to generate AMP 6. With the preliminary results of *in vitro* reconstitution, the saturation kinetic constants of NucV and *Sc*APRT were determined by the formation rates of AMP 6 at different concentrations of PRPP 5 or adenine 3 with the fixed concentration of excess adenine 3 or PRPP 5, respectively (Table 2).

The kinetic constants of NucV and *Sc*APRT for PRPP and adenine

**Table:** 

PRPP^a^			
Enzyme	*k*_cat_ (s^−1^)	*K*_m_ (μM)	*k*_cat_/*K*_m_ (μM^−1^ s^−1^)
NucV	1.34 ± 0.07	21.4 ± 3.0	0.062
*Sc*APRT	9.72 ± 0.47	9.8 ± 2.0	0.987

a The constants were determined at the fixed concentration of 500 μM adenine with varied PRPP catalyzed by 1 μM enzyme.

b The constants were determined at the fixed concentration of 500 μM PRPP with varied adenine catalyzed by 1 μM enzyme.

**Table:** 

Adenine^b^			
Enzyme	*k*_cat_ (s^−1^)	*K*_m_ (μM)	*k*_cat_/*K*_m_ (μM^−1^ s^−1^)
NucV	2.09 ± 0.09	6.8 ± 1.4	0.309
*Sc*APRT	16.59 ± 0.47	2.8 ± 0.6	5.863

**Fig. 5:**
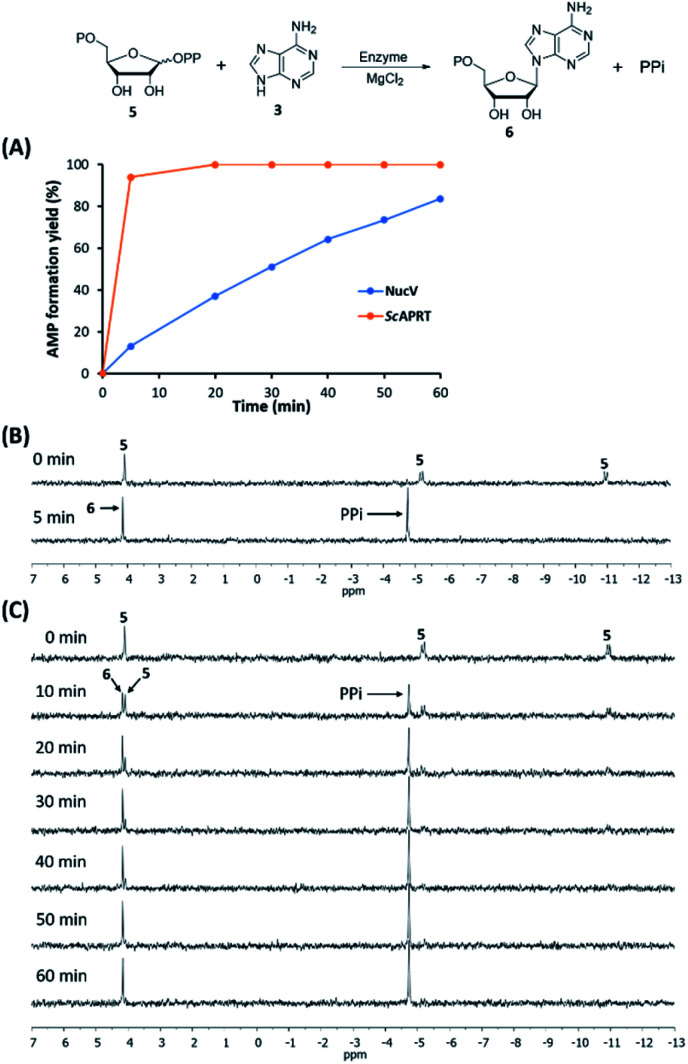
NucV catalyzed the reaction slower than *Sc*APRT under the same condition. The relative rate of AMP 6 formation was analyzed by HPLC (A). PPi formation and PRPP 5 consumption by *Sc*APRT (B) and NucV (C) were monitored by ^31^P-NMR.

In the Table 2, the apparent *k*_cat_(PRPP) and *k*_cat_(adenine) of NucV are both much lower than those of *Sc*APRT. The apparent *K*_m_(PRPP) and *K*_m_(adenine) of NucV are both higher than those of ScAPRT. These results suggested that NucV is far less efficient in AMP synthesis compared to *Sc*APRT. Then, we conducted time course experiments to monitor the rate of AMP formation by both enzymes in the *V*_max_ condition by HPLC analysis. In the designed condition, *Sc*APRT completed the reaction in 5 min, while NucV took about 1 hour (Fig. 5A). To confirm these results, ^31^P-NMR was utilized to monitor the formation of PPi (*δ* = −4.75 ppm) and the disappearance of PRPP 5 (*δ* = 4.09, −5.17, and −10.95 ppm) in the assays (Fig. 5B and C). The NMR analysis showed the same results. In order to confirm that the rapid AMP synthesis is a characteristic of APRT, *E. coli* APRT was also analyzed in the same condition, which is its *V*_max_ condition according to its kinetic constants (Table S2†). The amino acid sequence of *Ec*APRT has 70% similarity and 51% identity with *Sc*APRT. In the ^31^P-NMR analysis, *Ec*APRT also completed the reaction in 5 min (Fig. S7†). All experiments suggested that NucV catalyzed slower AMP formation than *Sc*APRT.

Although we can not show a direct evidence that NucV could catalyze 4′-F-PRPP 7 faster than PRPP 5, our indirect experiments showing that NucV acts relatively slowly on PRPP 5 support our hypothesis (Scheme 1). Additionally, the mRNA level of *nucV* transcript was reported to increase by 110 times with bldA complementation to rescue nucleocidin synthesis, suggesting the importance of NucV for nucleocidin production.

## 4. Conclusions and perspective

In this research, soluble MBP-NucPNP and NucV were successfully expressed and purified. The *in vitro* characterization suggested that NucPNP acts on MTA 2 or adenosine 4 to generate adenine 3 in presence of phosphate. Furthermore, NucV catalyzes slower AMP 6 formation from PRPP 5 and adenine 3 compared with *Sc*APRT, suggesting PRPP may not be its natural substrate.

Taking all information in account, the proposed nucleocidin biosynthetic pathway is shown in Scheme 1. NucPNP catalyzes adenine 3 formation from MTA 2. NucV could catalyze 4′-F-AMP 8 from 3 and 4′-F-PRPP 7. However, NucV also catalyzes AMP 6 formation. After dephosphorylation, adenosine 4 could be recycled to form adenine 3.

For nucleocidin biosynthesis, C–F formation is the most attractive step to investigate, but is still mysterious. NucJ has been proposed as a fluorinase candidate.^18^ We would suggest to test PRPP 5 for *in vitro* reconstitution of NucJ or any candidate proteins. We hope that our results and hypothesis can help researchers to solve the enigma of C–F formation in nucleocidin biosynthesis.

## Conflicts of interest

There are no conflicts to declare.

## Supplementary Material

RA-011-D0RA10878B-s001
