# Supplementary material for: Characterization of NucPNP and NucV involved in the early steps of nucleocidin biosynthesis in Streptomyces calvus
Source: RSC Adv. 2021 Jan 15;11(6):3510–5. doi: 10.1039/d0ra10878b (PMC8694150; doi:10.1039/d0ra10878b)
Supplement: RA-011-D0RA10878B-s001 [file RA-011-D0RA10878B-s001.pdf]

Electronic Supplementary Material (ESI) for RSC Advances.
This journal is © The Royal Society of Chemistry 2020

Characterization of NucPNP and NucV Involved in the Early Steps of Nucleocidin Biosynthesis in *Streptomyces calvus*

Supporting Information

Utumporn Ngivprom,‡[a] Surayut Kluaiphanngam,‡[a] Wenjuan Ji,[c] Siriwalee Siriwibool,[a] Anyanee Kamkaew,[a] James R. Ketudat Cairns,[a,b] Qi Zhang,[c]* and Rung-Yi Lai[a,b]*

[a] School of Chemistry, Institute of Science, Suranaree University of Technology, Nakhon Ratchasima, 30000 Thailand

[b] Center for Biomolecular Structure, Function and Application, Suranaree University of Technology, Nakhon Ratchasima, 30000 Thailand

[c] Department of Chemistry, Fudan University, Shanghai, 200433 China

‡ These authors contributed equally to this article.

MBP-NucPNP overexpression and purification

Six milliliters of overnight culture of *E. coli* SHuffle T7 Express *lysY* containing pET28-MBP-NucPNP was inoculated into 1 L cultures in Luria-Bertani broth (LB) with the final concentration of 50 µg/mL kanamycin. The culture was shaken at 200 rpm and 30 °C until OD_{600} reached about 0.6. The protein expression was induced by adding Isopropyl-β-D-1-thiogalactopyranoside (IPTG) with the final concentration of 200 µM. The culture mixture was shaken for an additional 16 hours at 200 rpm and 20 °C. The cells were spun down by centrifugation at 7,000 rpm and 4 °C for 20 minutes. The cells were resuspended in the lysis buffer (20 mM Tris-HCl, 200 mM NaCl, 1 mM EDTA, pH 7.4). The cells were lysed by sonication (1.5 s cycle, 50 % duty), followed by the centrifugation at 12,000 rpm and 4 °C for 40 minutes. The resulting cell lysate was clarified by centrifugation and the proteins were purified by amylose resin (New England Biolabs) following the manufacturer's instructions. After elution, the protein was desalted using a 10-DG column (BioRad) pre-equilibrated with 100 mM Tris-HCl buffer, 30% glycerol, pH 7.5. The purified protein was stored in aliquots at -80 °C.

Overexpression and purification of NucV, *Sc*APRT, and *Ec*APRT

Six milliliters of overnight culture of *E. coli* BL21(DE3) containing pET28-NucV, pET28-*Sc*APRT or pET28-*Ec*APRT was inoculated into 1 L cultures in Luria-Bertani broth (LB) with the final concentration of 50 µg/mL kanamycin. The culture was shaken at 200 rpm and 37 °C until OD_{600} reached about 0.6. The protein expression was induced by adding Isopropyl-β-D-1-thiogalactopyranoside (IPTG) with the final concentration of 200 µM. The culture mixture was shaken for an additional 16 hours at 200 rpm and 20 °C. The cells were spun down by centrifugation at 7,000 rpm and 4 °C for 20 minutes. The cells were resuspended in the lysis buffer (300 mM NaCl, 50 mM NaH_2PO_4, and 10 mM Imidazole). The cells were lysed by sonication (1.5 s cycle, 50 % duty), followed by the centrifugation at 12,000 rpm and 4 °C for 40 minutes. The resulting cell lysate was clarified by centrifugation and the proteins were purified on a Ni-NTA column (QIAGEN) following the manufacturer's instructions. After elution, the protein was desalted using a 10-DG column (BioRad) pre-equilibrated with 100 mM Tris-HCl buffer, 30% glycerol, pH 7.5. The purified protein was stored in aliquots at -80 °C.

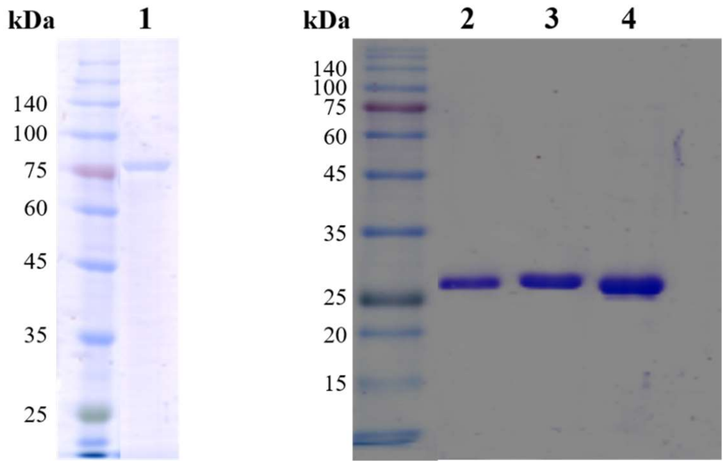

Figure S1. The SDS-PAGE analysis of MBP-NucPNP (#1), NucV (#2), *Sc*APRT (#3), and *Ec*APRT (#4).

HPLC condition for the analysis of assays

The following linear gradient, at a flow rate of 1 mL/min, on a ZORBAX Eclipse XDB-C18 (4.6 mm x 150 mm, 5 µm ID) was used: solvent A is 50 mM potassium phosphate (pH 6.6), solvent B is water, solvent C is methanol; 0 min: 100% A; 5 min: 90% A, 10% B; 10 min, 60% A, 25% B, 15% C; 12 min: 60% A, 25% B, 15% C; 14 min: 10% A, 30% B, 60% C; 16 min: 10% A, 30% B, 60% C; 19 min: 100% A; 24 min: 100% A.

The amino acid sequence of MBP-NucPNP (the underlined segment is NucPNP)

MGSSHHHHHHSSGLVPRGSHMASGKIEEGKLVIWINGDKGYNGLAEVGKKFEKDTGIKVTVEHPDKLEEKFPQVAAT
GDGPDIIFWAHDRFGGYAQSGLLAEITPDKAFQDKLYPFTWDAVRYNGKLIAYPIAVEALSLIYNKDLLPNPPKTWEEIP
ALDKELKAKGKSALMFNLQEPYFTWPLIAADGGYAFKYENGKYDIKDVGVDNAGAKAGLTFLVDLIKNKHMNADTDYS
IAEAAFNKGETAMTINGPWAWSNIDTSKVNYGVTVLPTFKGQPSKPFVGVLSAGINAASPNKELAKEFLENYLLTDEGL
EAVNKDKPLGAVALKSYEEELAKDPRIAATMENAQKGEIMPNIPQMSAFWYAVRTAVINAASGRQTVDEALKDAQTRI
TKGENLYFQGGGMTDLPRADIGVIGGSGLYSFLDDVTEVPVTTPYGPPSDALLVGEYAGRTIAFLPRHGRSHSVPPHRIN
YRANLWALRSVGVRRVLAPCAVGSLDAELGPGTLVVPDQVIDRTYGRENTYFDGLPREDGTFPPVAHAPMADPYCST
GRETVIATAREQGWPPHPEGTLVVIQGPRFSTRAESLWHRAAGGTVVGMTGQPEAALARELGLCYTSIALVTDLDAGA
ETGEGVTHEEVLAVFRQNIDRLRPLLTATIKNLPGEDACACPDAPDAEHV

The amino acid sequence of NucV fusion protein (the underlined segment is NucV)

MSSHHHHHHSSGENLYFQGGGMTAPHQPPAADLGQPAAGPGGHLAGHIRDVVDHPRPGVTFKDITPLLADPGAFA
DTVDILSAMCTRLGATRIAGLEARGFLLAAPVALRCGAGCVPVRKAGKLPGETFSRAYELEYGTATLEIQRDAFRPEDRV
VVVDDVLATGGTAEAAIELVHSTGARVTGVVVLMELTFLPGRERLERLVKSDCVQAAIAV

The amino acid sequence of *Sc*APRT fusion protein (the underlined segment is *Sc*APRT)

MSSHHHHHHSSGENLYFQGGGMTELTDLSTLLLSRIRDVADHPEPGVMFKDITPLLADPAAFSALSDALADIARDTGAT
KVVGLEARGFILGAPAAVRAGVGFIPVRKAGKLPGATLSQAYDLEYGSAEIEVHAEDLSAGDRVLVVDDVLATGGTAEA
SLQLIRRAGAQVAGLAVLMELGFLGGRARLEPALAGAPLKALLTI

The amino acid sequence of *Ec*APRT fusion protein (the underlined segment is *Ec*APRT)

MSSHHHHHHSSGENLYFQGGGMTATAQQLEYLKNSIKSIQDYPKPGILFRDVTSLMEDPKAYALSIDLLVERYKNAGITK
VVGTEARGFLFGAPVALGLGVGFVPVRKPGKLPRETISETYDLEYGTDQLEIHVDAIKPGDKVLVVDDLLATGGTIEATVK
LIRRLGGEVADAAFIINLFDLGGEQRLEKQGITSYSLVPFPGH

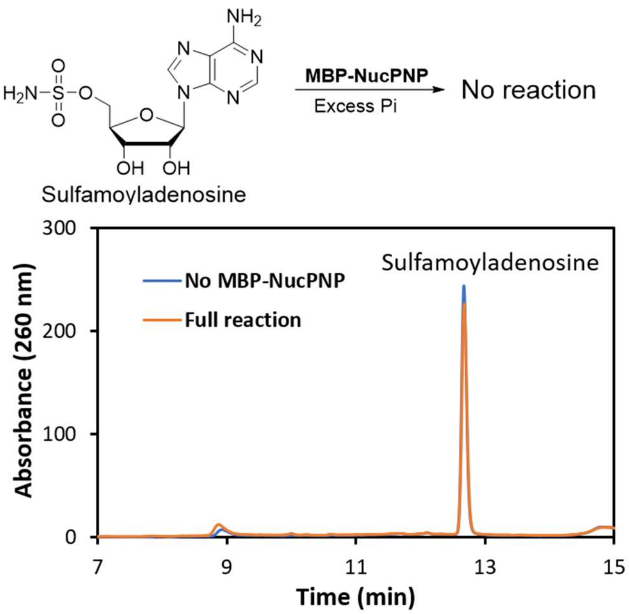

Figure S2. HPLC analysis of sulfamoyladenosine assayed with MBP-NucPNP to show no reaction.

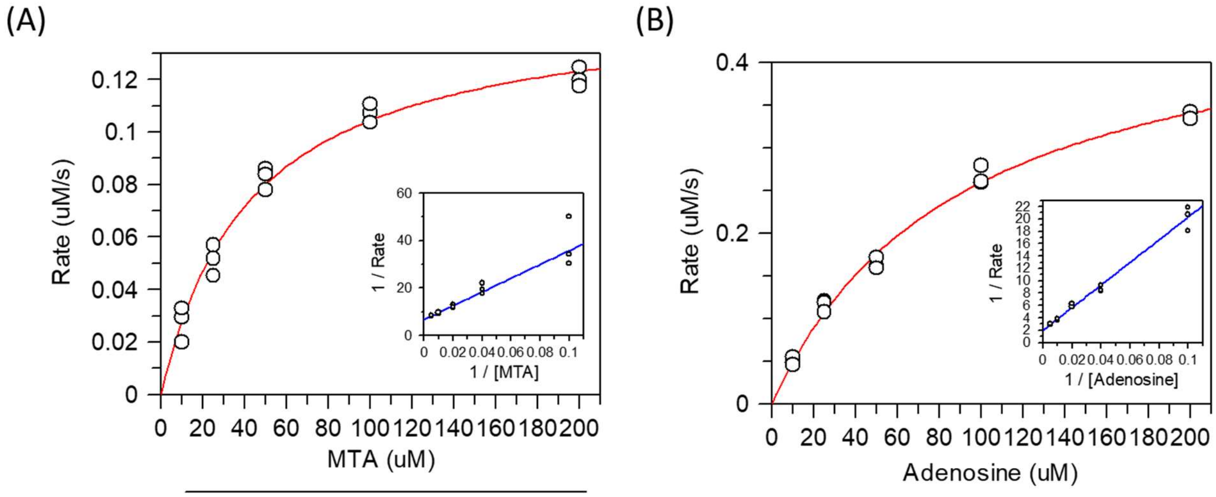

(A)

Parameter	Value	Std. Error
Vmax	0.1498	0.0051
Km	43.5348	4.1284

(B)

Parameter	Value	Std. Error
Vmax	0.4939	0.0217
Km	90.2983	8.1788

Figure S3. The Michaelis-Menten curves of phosphorylation of MTA (A) or adenosine (B) catalyzed by MBP-NucPNP.

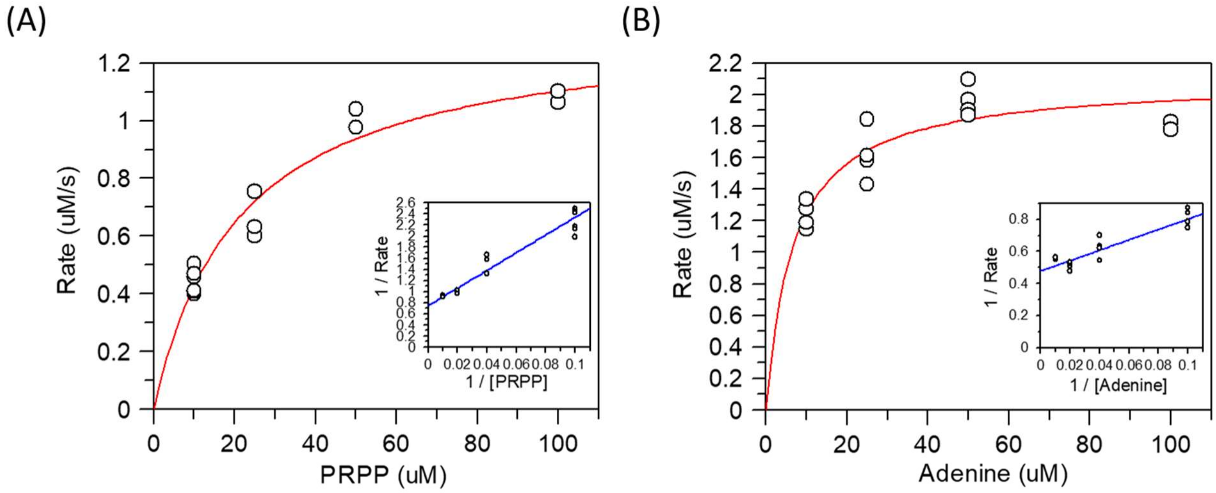

Parameter	Value	Std. Error
Vmax	1.3380	0.0746
Km	21.3628	3.0119

Parameter	Value	Std. Error
Vmax	2.0916	0.0912
Km	6.7701	1.3715

Figure S4. The Michaelis-Menten curves of AMP formation catalyzed by NucV.

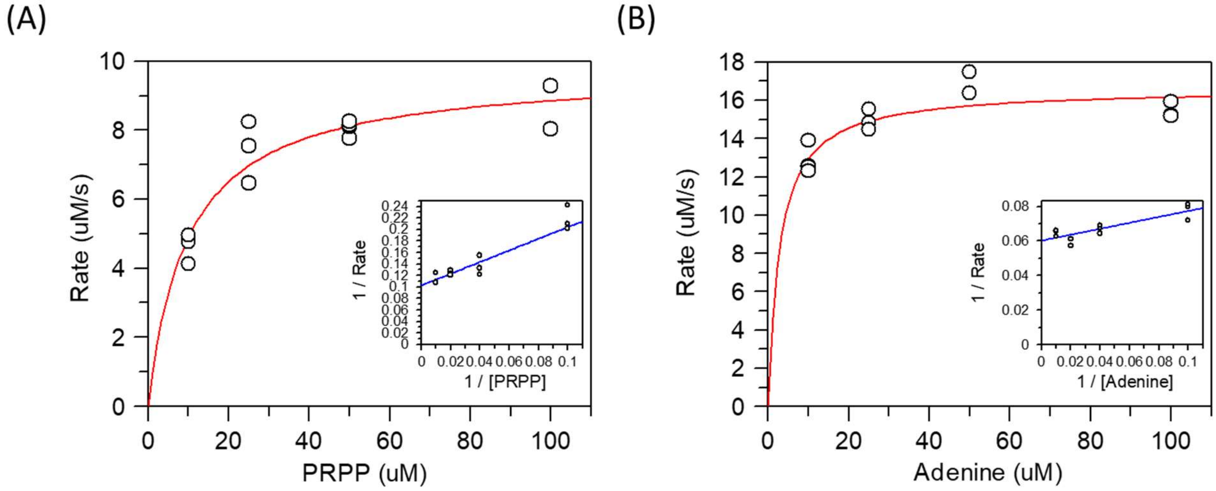

Parameter	Value	Std. Error
Vmax	9.7195	0.4744
Km	9.8473	1.9775

Parameter	Value	Std. Error
Vmax	16.5935	0.4709
Km	2.8300	0.6324

Figure S5. The Michaelis-Menten curves of AMP formation catalyzed by *Sc*APRT.

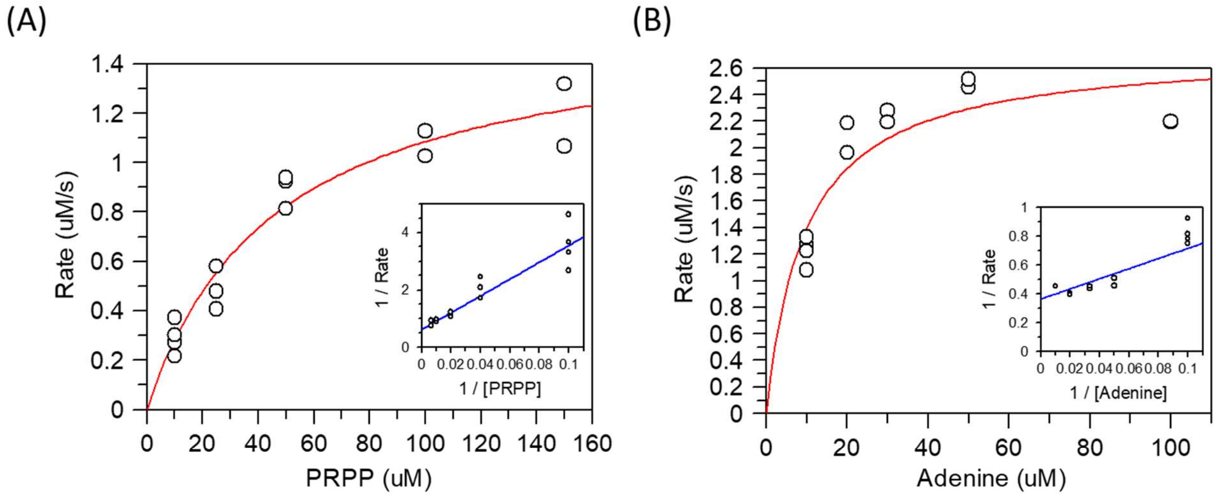

Parameter	Value	Std. Error
Vmax	1.5886	0.1285
Km	46.3869	9.0024

Parameter	Value	Std. Error
Vmax	2.7347	0.1934
Km	9.6313	2.4847

Figure S6. The Michaelis-Menten curves of AMP formation catalyzed by *Ec*APRT.

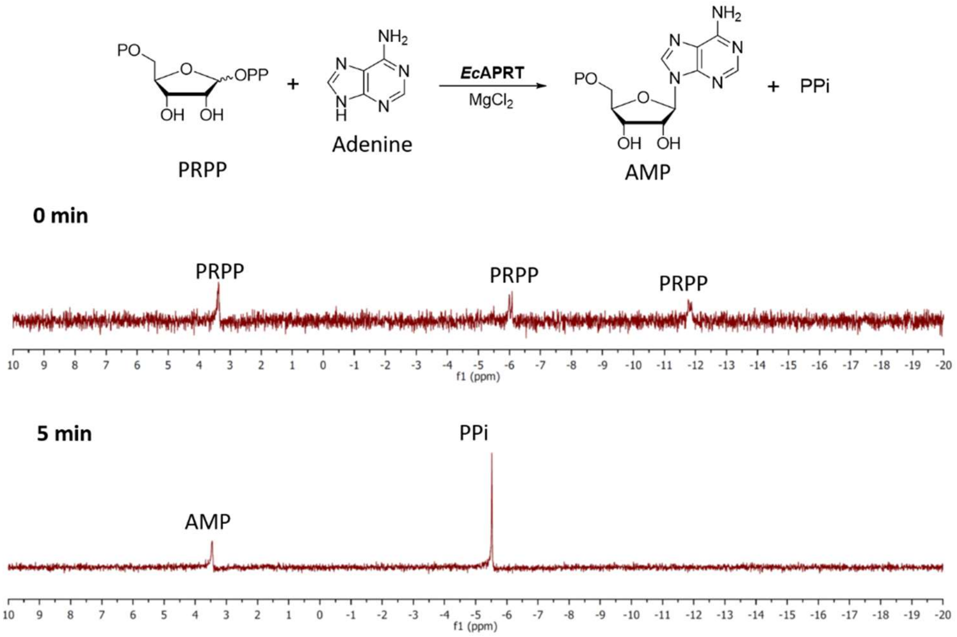

Figure S7. ^{31}P-NMR analysis of *Ec*APRT catalyzing AMP formation. The reaction rate was similar with that of *Sc*APRT (Fig. 5B).

Table S1. The increase in mRNA after the complement with a functional *bldA* reported by Zechel's group[1]

Gene	Predicted function	Fold of mRNA change*
nucV	Adenine phosphoribosyltransferase	110
nucPNP (*orf206*)	Purine nucleoside phosphorylase	151
nucJ	Radical SAM enzyme	85
nucGS	Glycosyltransferase	12

* mRNA increasing level with a functional *bldA* to restore nucleocidin production.

Table S2. The kinetic constants of *Ec*APRT for PRPP and adenine[a]

Substrate	k_{cat} (s^{-1})	K_m (μM)	k_{cat}/K_m ($\mu M^{-1}\ s^{-1}$)
PRPP	15.9 ± 1.3	46.4 ± 9.0	0.34
Adenine	27.3 ± 1.9	9.6 ± 2.5	2.84

[a] The k_{cat}, K_m, and k_{cat}/K_m were determined at the fixed concentration of 500 μM adenine with varied PRPP or 500 μM PRRR with varied adenine catalyzed by 0.1 μM of *Ec*APRT.

Synthesis of sulfamoyladenosine (4)

High resolution mass spectrometry (HR-MS) analysis was performed using a Q-Exactive™ Focus Hybrid Quadrupole-Orbitrap MassSpectrometer (Thermo Fisher) equipped with a Dionex Ultimate 3000 HPLC system (Thermo Fisher). The ^{1}H-NMR spectra were obtained on a Brucker 400 MHz NMR Fourier transform spectrometer. NMR spectra was recorded deuterated chloroform ($CDCl_3$) with residual chloroform (δ = 7.26ppm for ^{1}H-NMR) and TMS (δ = 0 ppm for ^{1}H-NMR) as the standard.

The reagent grade chemicals were purchased from Sigma or Energy Chemical without further purification. Purification of the product was carried out by preparation TLC (Thin layer chromatography) coated with GF254 silica gel and CMC, Carboxymethylcellulose sodium). For TLC analysis, precoated plates (HSGF254 silica gel) were used.

1. Preparation of 2′,3′-*O*-isopropylideneadenosine (2)

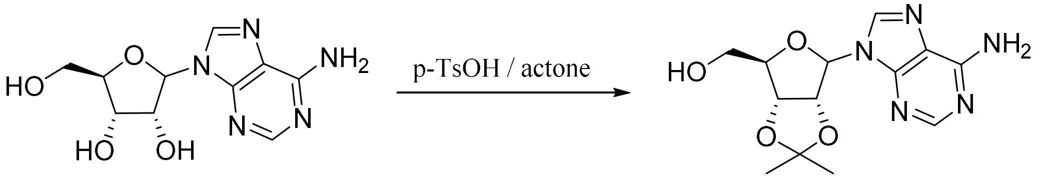

(1) **(2)**

The 2’,3’-*O*-isopropylideneadenosine **(2)** was prepared according to the procedure reported previously[2]. To a suspension of adenosine **(1)** (2.67 g, 10.0 mmol) in dry acetone (500 mL) was added *p*-TsOH monohydrate (19.0 g, 100.0 mmol) in one portion. The mixture was stirred at room temperature for 3 hours, and ice-cold saturated $NaHCO_3$ solution was added to the reaction with stirring over 5 minutes to adjust to pH 8.0. The volatiles were removed under reduced pressure and the remaining aqueous layer was extracted three times with 300 mL of ethyl acetate. The combined ethyl acetate layers were washed with brine and dried over anhydrous sodium sulfate. The ethyl acetate was removed under reduced pressure, and the product **(2)** was obtained as a white solid (93%): HR-ESI-MS ($[M+H]^+$): 308.1359 (calculated), 308.1402 (found). ^{1}H-NMR (400 MHz, CD_3OD): δ = 1.40 (s, 3H), 1.67 (s, 3H), 3.82 (d, 1H, *J* = 12.0 Hz), 4.00 (d, 1H, *J* = 12.0 Hz), 4.57 (s, 1H), 5.13 (d, 1H, *J* = 4.0 Hz), 5.23 (t, 1H, *J* = 8.0 Hz), 5.88~5.89 (m, 2H), 6.47 (brs, 2H), 7.88 (s, 1H), 8.35 (s, 1H).

2. Preparation of 2′,3′-*O*-isopropylideneadenosine-6’-*O*-amino sulfamate (3)

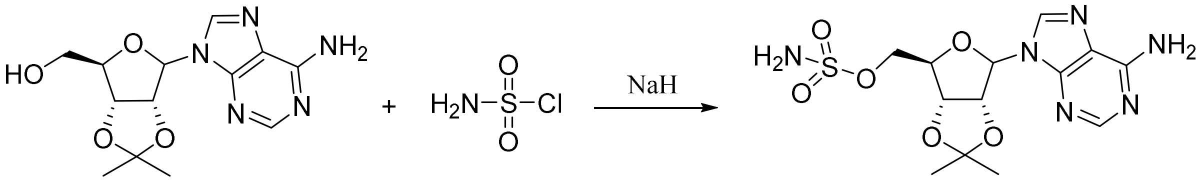

(2) **(3)**

The 2’,3’-*O*-isopropylideneadenosine-6’-*O*-amino sulfamate **(3)** was prepared according to the procedure reported previously with some modifications[3]. Under an atmosphere of nitrogen, the 2’,3’-*O*-isopropylideneadenosine **(2)** (307mg, 1 mmol, 1.0 eq) was dissolved in 1.5 mL THF and 1.5 mL NMP. The resulting solution was cooled in an ice-water bath and 60% NaH (78mg, 1.5 mmol, 1.5 eq) was added. The reaction mixture was stirred for 30 minutes with continuous cooling. Next, the sulfamoyl chloride (172.5 mg,1.5 eq in 1 mL NMP) was added dropwise, the reaction mixture was allowed to warm to room temperature and then stirred for 2 hours. Once the reaction was complete, the reaction mixture was once again cooled in an ice bath and any residual NaH was quenched through the dropwise addition of methanol. The reaction mixture was then concentrated in a rotary evaporator. The resultant residue was dissolved in water and extracted three times with 30 mL of ethyl acetate. The combined ethyl acetate layers were washed with brine and dried over anhydrous sodium sulfate. The ethyl acetate was removed in a rotary evaporator, and the crude product yellow oil (200mg) was obtained. HR-ESI-MS ($[M+H]^+$): 387.1087 (calculated), 387.1067 (found). The yellow oil was not purified and used directly in the next step.

3. Preparation of sulfamoyladenosine (4)

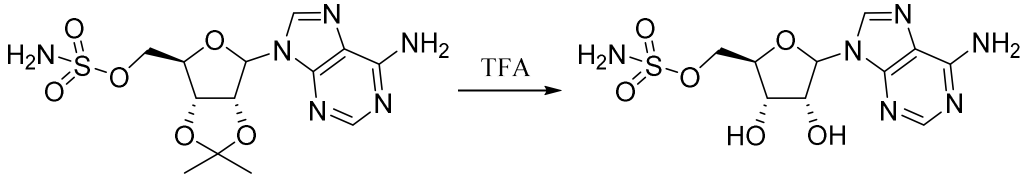

(3) (4)

The adenosine-6'-*O*-amino sulfamate **(4)** was prepared according to the procedure reported previously with some minor modifications[3]. To a round-bottom flask containing the crude product of sulfamoyladenosine **(4)** (200 mg yellow oil, 1.0 eq) was added TFA (8.0 ml) and distilled water (4.0 ml). The reaction mixture was stirred overnight at room temperature. The water and TFA were then removed using a rotary evaporator and the product was purified by silica gel chromatography with the mobile phase consisted of (CH_2Cl_2/MeOH/NH_4OH (9/1/1)). 94.5 mg grey slurry was obtained. The product contained NH_4OH as an impurity, and the amount ratio of NH_4OH and the product is about 3:1 from the ^{1}H-NMR spectrum. HR-ESI-MS ($[M+H]^+$): 347.0774 (calculated), 347.0764 (found). ^{1}H-NMR(DMSO-d_6, 400MHZ):δ = 4.10~4.29 (m, 3H), 4.61 (dd, 2H, J_1 = 5.2 Hz, J_2 = 5.2Hz), 5.45 (d, 1H, J = 5.6 Hz), 5.63 (d, 1H, J = 5.6 Hz), 5.92 (d, 1H, J = 5.2 Hz), 7.17 (brs, 2H), 7.31 (brs, 2H), 8.14 (s, 1H), 8.29 (s, 1H).

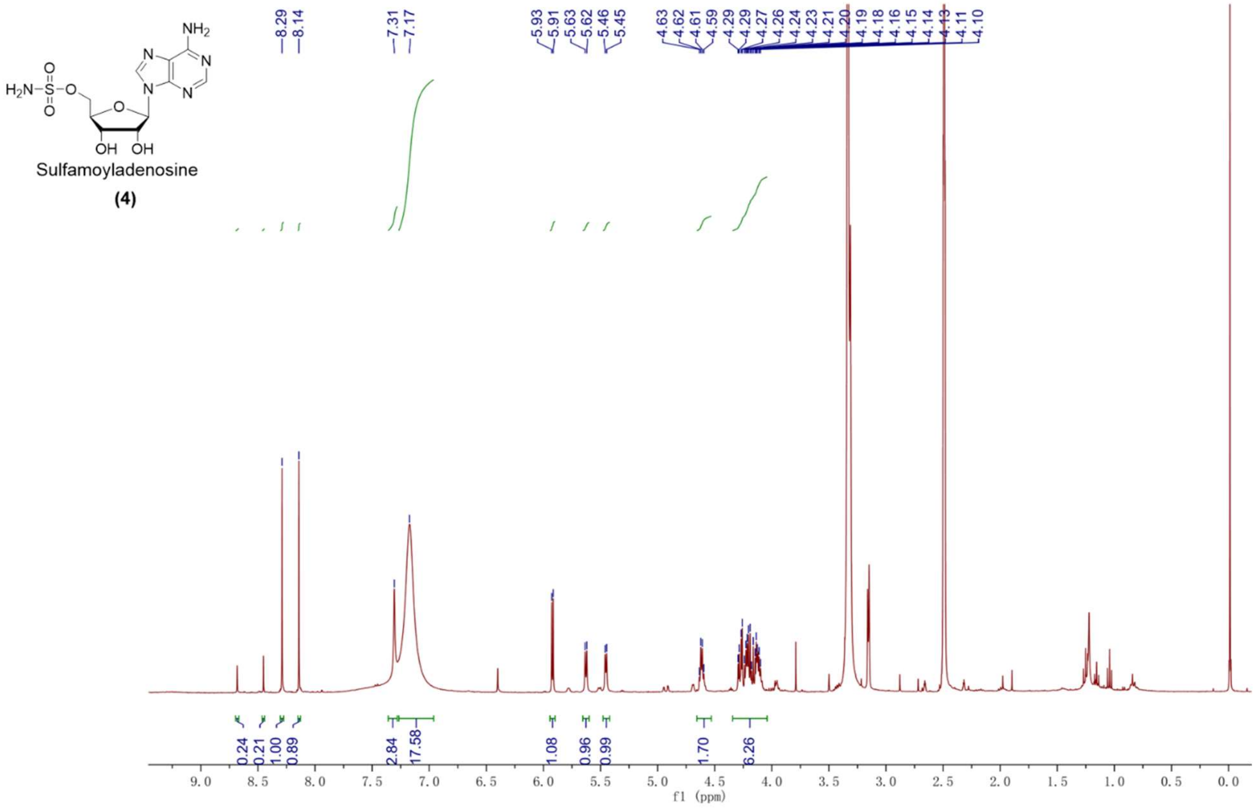

Figure S5. ^{1}H-NMR spectrum of sulfamoyladenosine **(4)**.

Reference

1 X. M. Zhu, S. Hackl, M. N. Thaker, L. Kalan, C. Weber, D. S. Urgast, E. M. Krupp, A. Brewer, S. Vanner, A. Szawiola, G. Yim, J. Feldmann, A. Bechthold, G. D. Wright and D. L. Zechel, *ChemBioChem*, 2015, **16**, 2498–2506.

2 W. Ji, X. Ji, Q. Zhang, D. Mandalapu, Z. Deng, W. Ding, P. Sun and Q. Zhang, *Angew. Chemie Int. Ed.*, 2020, **59**, 8880–8884.

3 J. L. Lukkarila, S. R. da Silva, M. Ali, V. M. Shahani, G. W. Xu, J. Berman, A. Roughton, S. Dhe-Paganon, A. D. Schimmer and P. T. Gunning, *ACS Med. Chem. Lett.*, 2011, **2**, 577–582.
